# Case Report: Psychopathological Syndromes in the Course of Lupus Erythematosus and the Co-occurrence of Lupus Erythematous With Mental Disorders

**DOI:** 10.3389/fpsyt.2021.668050

**Published:** 2021-06-24

**Authors:** Ewa Stelmach, Jolanta Masiak

**Affiliations:** Second Department of Psychiatry, Medical University of Lublin, Lublin, Poland

**Keywords:** systemic lupus erythematosus, bipolar disorder, affective and psychotic disorders, steroid-induced mood and psychotic disorders, neuropsychiatric systemic lupus erythematosus

## Abstract

**Background:** Systemic lupus erythematosus (SLE) is an autoimmune disease that leads to a chronic inflammatory process in tissues and organs. The neuropsychiatric systemic lupus erythematosus (NPSLE) is a set of neuropsychiatric symptoms that derive from the central and peripheral nervous system and are observed in the course of SLE.

**Case Report and Final Diagnostic and Therapeutic Results:** A diagnostic and therapeutic process in a patient with the signs and symptoms of SLE and bipolar disorder (BD) has been described. Bipolar disorder has been diagnosed as a primary disorder while SLE as a comorbid disease.

**Discussion:** Common immunological mechanisms in BD and SLE are the reason for difficulties in diagnosing BD with co-occurring SLE. It should be determined whether BD is a primary disorder or a secondary component of a clinical picture of SLE (NPSLE) or whether mood and/or psychotic disorders are the result of steroid therapy in the course of SLE (steroid-induced mood and psychotic disorders, SIMPD).

**Conclusion and the Patient's Perspective:** The presented case report is a unique description of a patient with a primary diagnosis of BD with comorbid SLE.

## Introduction

The systemic lupus erythematosus (SLE) was probably first described in 1851 by Cazenave. It is an autoimmune disorder that leads to chronic inflammation in tissues and organs (1).

### Epidemiology

The prevalence of SLE is estimated at 40–50 in 100,000 persons and varies depending on the population studied: in North America, it equals to 241 in 100,000 persons, while in Australia to 0 in 847 persons. Similarly, the incidence rate varies and is equal to 23.2/100,000 persons in North America and 0.3/100,000 persons in Africa and Ukraine (2, 3).

Studies indicate an average of 10 times the incidence of SLE in women compared to men (with a ratio of 2:1 to 15:1) (3), and the results of the studies of Somers et al. on the British population indicate a higher incidence of SLE in women than in men (7.89/100,000 vs. 1.53/100,000 persons) (4). Also, peak incidence varies by gender: in women, it is between the third and seventh decade of life, while in men between the fifth and seventh decade of life (3).

### Etiology

The following factors are considered in the SLE etiology:

1. genetic factors: 20 genetic variants on more than 10 chromosomes have been described and the disease compatibility among monozygotic twins is estimated at 14–57%,
2. hormonal factors: a more frequent incidence in women of reproductive age indicates the estrogen involvement,
3. environmental factors: sunlight, retroviruses, Epstein-Barr virus (EBV),
4. complex immunological disorders: the presence of antinuclear antibodies (1, 5–7).

### Diagnostic Criteria

The diagnostic criteria for SLE have been modified over time. The first version comes from 1982 and was prepared by American College of Rheumatology (ACR). The revised version was prepared in 1997 and was prompted by a better understanding of the nature of the disease—skin symptomatology of SLE as well as certain clinical syndromes were described, and the immune tests became a part of the clinical practice. The criteria developed by the Systemic Lupus International Collaborating Clinics (SLICC) in 2012 included mucosal and skin changes as well as neuropsychiatric disorders. Currently, the diagnosis of SLE is based on the diagnostic criteria of the European League Against Rheumatism (EULAR) and ACR (8, 9).

### Symptomatology

Clinical signs and symptoms of SLE are associated with a chronic inflammatory process in tissues and organs and affect all systems (10).

### Drug-Induced Lupus

Another issue is drug-induced lupus (DIL) that appears secondary to pharmacotherapy and affects 15,000–30,000 people in the US annually. Since 1945 over 90 drugs associated with DIL have been identified (11). Its pathogenesis is associated with the genetic predisposition and the individual course of drug metabolism.

Hydralazine and procainamide are the two drugs with the highest DIL risk (for procainamide the risk is 30%, for hydralazine 5–10%). Several groups of drugs that can induce DIL have been identified: 1. anti-TNF-alpha drugs e.g., etanercept, infliximab, 2. antifungal drugs e.g., terbinafine, antibiotics e.g., minocycline, and antimycobacterial drugs e.g., isoniazid, pyrazinamide, rifabutin, 3. antiepileptic drugs—phenytoin, valproate, carbamazepine, 4. Anti-malaria drugs e.g., quinidine, and 5. antihypertensives e.g., minoxidil, timolol, 6. antiarrhythmic agents e.g., procainamide, propafenone, 7. antihistaminic agents e.g., hydroxyzine (12).

### Neuropsychiatric Lupus

Neuropsychiatric systemic lupus erythematosus (NPSLE) is a set of neuropsychiatric signs and symptoms which come from the central nervous system (CNS) and are described in the course of SLE. They are present among around 56% of patients with SLE. Among the patients diagnosed with NPSLE, 90% experience psychiatric disorders associated with a dysfunction of CNS (2).

The ACR has differentiated 19 neuropsychiatric syndromes in the course of NPSLE, 12 of which are associated with the CNS disorders (headaches, seizures, cerebrovascular disease, demyelination syndrome, myelopathy, motor disorders, consciousness disorders) and 7 with the peripheral nervous system disorders (mononeuropathy, polyneuropathy, cranial neuropathy, Guillain-Barre syndrome, plexopathy, autonomic system disorders, myasthenia gravis). For NPSLE diagnosis, a patients should meet SLE criteria, present typical neuropsychiatric symptoms, and other possible causes should be excluded (based on clinical status, laboratory tests, neuroimaging, CSF analysis) (13).

The prevalence of psychiatric symptoms in the course of SLE was studied by Fernandez et al. Among 85 patients with the diagnosis of SLE (94% of whom were women) the most common were: cognitive function disorders (43.51%), anxiety disorders (35.41%), affective disorders (34.40%), psychotic disorders (1.1%) (14).

**Figure 1:**
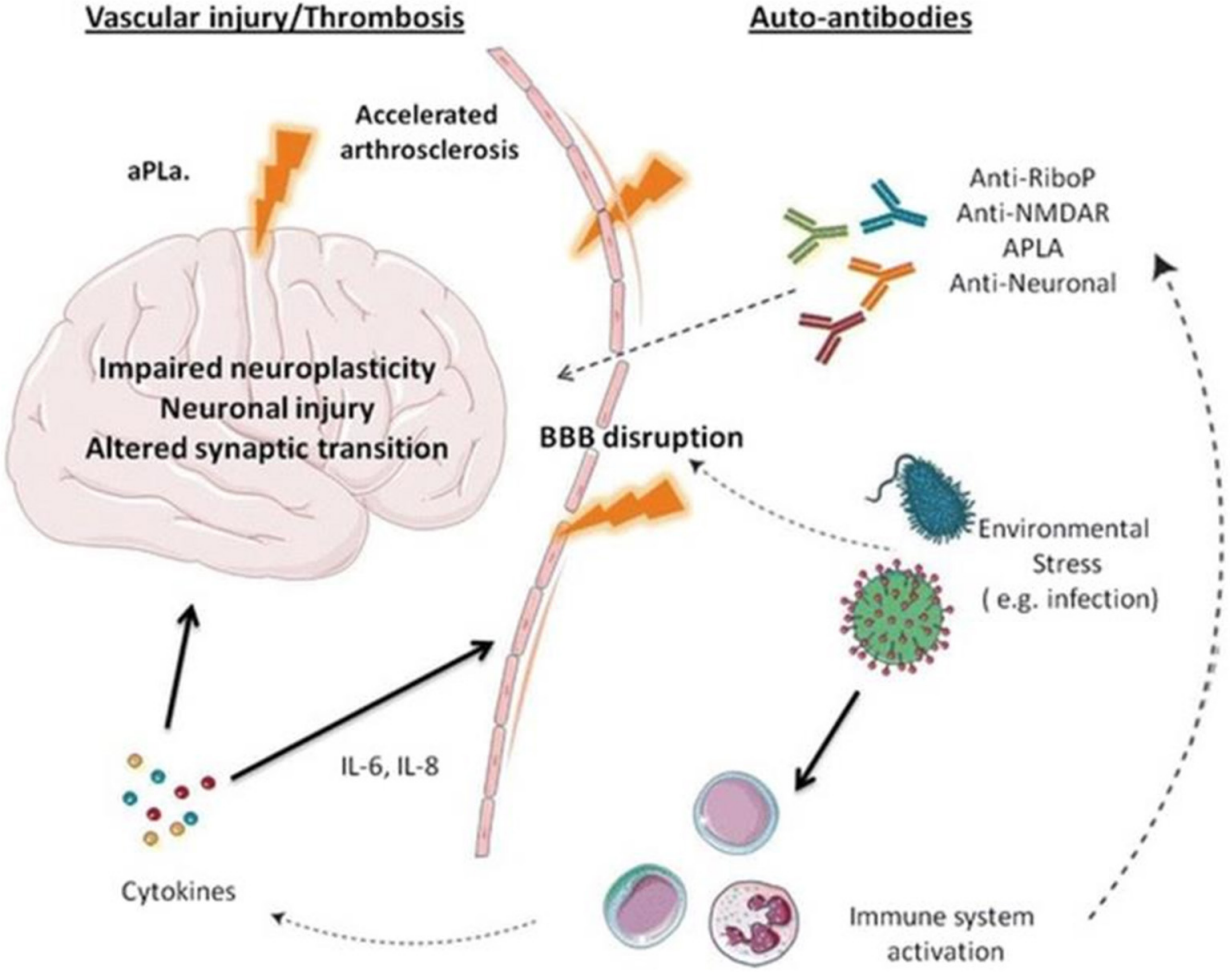
Pathogenesis of NPSLE.

The pathogenesis of NPSLE is presented in Figure 1 (15).

In this model, autoantibodies that have been identified as more than 20 and proinflammatory cytokines, i.e., interleukins−2, 6, 8, 10 (Il-2, Il-6, Il-8, Il-10), interferon-alpha, and gamma are the factors leading to a disruption of the blood-brain barrier (BBB). Among well-recognized autoantibodies, we include anti-ribosomal P antibody, anti-DNA/NR2 antibody, anti-DNA-6 antibody, anti-DNA/16-6 idiotype (monoclonal), antiphospholipid and anticardiolipin antibodies, and anti-GABA antibodies. Moreover, the BBB disruption is also due to environmental factors, i.e., infections, stress, hypoxia, and additionally, the changes in the CNS may appear due to small vessel homeopathy, which leads to the disruption of neuroplasticity, the disruption in neurons, and synaptic transmission (15, 16).

The NPSLE diagnostics is based on the examination of cerebrospinal fluid (CSF), in which immunological markers are detected. The biomarkers used in NPSLE diagnostics include plasminogen activator inhibitor-1, metalloproteinase-9 (MMP-9), proteins i.e., S100B, S100A8/9, lipocalin associated with neutrophil gelatinase and anti-ribosomal P and anti-DNA antibodies.

The neuroimaging tests have an additional function here: head computed tomography (CT) examination enables to exclude focal lesions, i.e., stroke, hemorrhage, tumor, and head magnetic resonance imaging (MRI) examination reveals neuritis and cerebral vascular lesions in 80–90% of patients. However, over 50% of patients show no deviations in the head MRI (2).

### Treatment

Systemic lupus erythematosus treatment is based on high-dose steroid therapy with immunosuppressive cyclophosphamide, which prevented dendritic cells atrophy on the mouse model and with monoclonal antibodies against B-cells (rituximab), proinflammatory anti-CD20 cytokines (belimumab), Il-6 (tocilizumab), costimulatory particles (abatacept), complement system (eculizumab). Plasmapheresis and anticoagulants are also used, and the future of SLE therapy includes drugs against the p40 subunit of IL-12/23 (17, 18).

### Methods

Written informed consent was obtained from the individual for the publication of any potentially identifiable images or data included in this article.

## Case Report

A 47-year-old female with a university degree (orthodontist), married, living with her son and husband, professionally active has been admitted to the II Clinic of Psychiatry and Psychiatric Rehabilitation in Lublin due to tension, irritability, circumstantial thinking and tangentiality, racing thoughts, elevated mood and increased psychomotor drive, difficulties in concentrating, grandiose attitude, personal, professional, and social dysfunctions, verbal aggression, lack of criticism, and insight. It was her fourth psychiatric hospitalization.

On admission, mental health examination revealed the patient was of clear awareness, had normal autopsychic and allopsychic orientation, she presented dysphoric mood, circumstantial speech, tangentiality, loquaciousness, increased psychomotor drive.

The patient was treated psychiatrically from 2014 to 2015 with the diagnosis of bipolar disorder (BD), and since 2016 she has been treated for SLE.

The first psychiatric hospitalization was in September 2014. Previously, the patient was hospitalized in the Infectious Diseases Hospital in which she was treated with the suspicion of yersiniosis, manifested with peripheral arthritis. The pulses of Solu-Medrol, as well as Encorton p.o., were administered. After discharge, there was a sudden change of behavior with euphoria, insomnia, the patient was sharing referential religious thoughts. Moreover, she presented unexpected behaviors. In the Psychiatry and Neurology Institute in Warsaw, olanzapine 10 mg/d and valproate 600 mg/d were administered, which resulted in partial mental state improvement.

The patient checked out of the hospital at her request, against medical recommendations, with a diagnosis of an episode of mania with signs and symptoms of psychosis in the course of steroid therapy. Due to her lack of consent for treatment, she was discharged from the hospital, without signs and symptoms of severe psychosis, with moderate signs and symptoms of mania and with recommendations to continue the treatment in ambulatory conditions. After the discharge, the patient discontinued pharmacotherapy.

The second hospitalization was in June/July 2015 in the Institute of Psychiatry and Neurology in Warsaw with the diagnosis of psychotic mania in the course of BD. The patient was referred by a psychiatrist from a mental health clinic and was brought in the ambulance. The deterioration of the patient's mental health condition was initially manifested with agitation and insomnia, then there was deterioration in contact, disorganized behavior, the patient refused to drink liquids or eat, and had religious delusions. The patient had been complaining of joint pain, weakness, chronic cough and lower limbs swelling for over a year. During the hospitalization, an elevated level of antinuclear antibodies, as well as axillary lymphadenopathy, were found. The diagnostics toward connective tissue diseases was recommended. The results of serological examinations performed during the hospitalization are shown in Table 1.

**Table 1:** Serological examinations.

**Antibody**	**Titer**	**Reference**
ANA (nuclear pattern)	1:640	1:40
ANA (against cytoplasm)	1:2,560	1:40
cANCA	Negative	
pANCA	Negative	
aCCP	Negative	

Valproic acid 1,500 mg/d and olanzapine 15mg/d were administered with a satisfactory effect. Soon after the discharge, the patient discontinued pharmacotherapy.

The patient's third psychiatric hospitalization at the Psychiatry and Neurology Institute in Warsaw in August/September 2016 was due to the relapse of affective and psychotic disorders: the patient had insomnia, was agitated, irritable, verbally aggressive toward her husband, limited the intake of food and liquids, and had various persecutory and grandiose delusions. She was loquacious, had racing thoughts and circumstantial speech. Moreover, during the patient's stay, the symptoms of arthritis of variable intensity were observed, the patient reported radiating joints pain. The treatment included zuclopenthixol and valproate, as well as clorazepate and zopiclone as needed. Lithium carbonate was also administered with a recommendation to increase the dose up to a therapeutic level and to control the lithium serum concentration in the ambulatory conditions. After the mental state stabilization, the patient was discharged with the diagnosis of psychotic mania in the course of BD. After the discharge the patient took lithium carbonate and valproate for few years until March 2019.

The first hospitalization in the Rheumatology Clinic was in October 2016 due to a several-year-history of lymphadenopathy, recurrent subfebrile state, skin hyperaesthesia, paresthesia of the fingers of both hands. In laboratory tests: periodically elevated inflammatory parameters (OB > CRP), anemia, decreased concentration of C3 and C4 components of the complement system, the test for anti-dsDNA antibodies: positive, antinuclear ANA antibodies: titer 1:1,280 (grainy fluorescence). The level of anti-ribosomal P antibody 5,000 times exceeded the norm and there were no findings in neuroimaging. The patient was discharged from the hospital with the diagnosis of SLE.

The patients' following hospitalizations in the Rheumatology Clinic in November 2016 and December 2017 were due to the need for intravenous steroid therapy. After the discharge, mycophenolate mofetil and hydroxychloroquine were administered in ambulatory conditions. After the discharge from the Rheumatology Clinic in 2017 the patient systematically took mycophenolate mofetil and hydroxychloroquine until January 2020.

## The Final Diagnostic and Therapeutic Results

During the fourth psychiatric hospitalization in the period from 02.01.2020 to 13.02.2020, the patient was consulted with a rheumatologist—the continuation of the previous treatment was recommended. The patient denied exacerbation of clinical (somatic) symptoms of SLE. In laboratory tests: slight anemia without cytopenia, without the signs and symptoms of kidney dysfunction, the C3 and C4 components of the complement system were normal, and anti-dsDNA antibodies were negative. The patient was discharged in a stable condition with the diagnosis of a moderate episode of mania in the course of BD as well as comorbid SLE and was recommended pharmacological treatment: olanzapine 20 mg/d, quetiapine 400 mg/d, carbamazepine 600 mg/d, and injections with a sustained-action aripiprazole 400 mg/month due to lack of a patient cooperation in past. Pharmacological treatment due to psychiatric indications is shown in Table 2. In the SLE treatment, mycophenolate mofetil 1,500 mg/d and hydroxychloroquine 400 mg/d were continued, without steroid therapy. The diagnosis of primary BD was set due to typical symptoms of BD and lack changes in laboratory tests as well as in clinical, somatic status of the patient.

**Table 2:** Pharmacological treatment during fourth psychiatric hospitalization.

**Drug/method**	**Max. dose**	**Period**	**Effect**	**Side effects**
Sodium valproate	1,200 mg/day	02.01.2020–10.01.2020	Discontinued	Rash on the skin of face and cleavage
Quetiapine	400 mg/day	02.01.2020–13.02.2020	Satisfactory	Not observed
Aripiprazole	30 mg/day	07.01.2020–13.02.2020	Satisfactory	Not observed
Levomepromazine (as a hypnotic, PRN)	25 mg/day	07.01.2020–12.01.2020	Satisfactory	Not observed
Carbamazepine	600 mg/day	11.01.2020–13.02.2020	Satisfactory	Not observed
Sustained-action aripiprazole	400 mg/month ampule, i.m.	12.02.2020	Satisfactory	Not observed

## Discussion

Common immunological mechanisms in BD and SLE are the reasons for difficulties in the diagnosis of BD with comorbid SLE.

One should consider whether BD is a primary disorder or whether it is a secondary element of the clinical picture of SLE (NPSLE) or whether the affective and/or psychotic disorders are the result of steroid therapy in the course of SLE (steroid-induced mood and psychotic disorders SIMPD) (19, 20).

Spiegel et al. indicated similar changes in the CNS in the course of BD and SLE: the changes in the hippocampus and amygdaloid bodies, immune system disorders, increased levels of proinflammatory cytokines (21).

The neuro-immunological factors described in the BD pathogenesis are a weakened response to mitogens, a weakened NK cells activity, a lower absolute count of B and T lymphocytes, increased levels of C-reactive protein (CRP), alpha-1-acid glycoprotein (AGP), alpha-chymotrypsin (ACT) as well as increased levels of proinflammatory cytokines IL-1, IL-6, increased secretion of corticoliberin (CRH), hypercortisolism, elevated levels of soluble interleukin-2 receptor (mania), and viral infections (herpes, Borna, parvovirus P19) (22–24).

Studies indicate an increased susceptibility of patients with SLE to BD. Tiosano et al. examined 5,018 persons with SLE and 25,090 healthy ones and the results indicate a higher incidence of BD in SLE patients than in the healthy control group (BD percentage 0.62% vs. 0.26%, *p* < 0.001) (25).

Similarly, Wang et al. examined 65,498 subjects with systemic autoimmune diseases and 261 992 subjects from the control group and found an increased risk of BD incidence in patients with SLE, as well as with rheumatoid arthritis (RA), autoimmune vasculitis, Sicca syndrome, and Crohn disease (26).

Perez et al. described the case of a 59-year-old female with the history of BD who was hospitalized due to a probable drug overdose (trazodone, acetaminophen, butalbital, caffeine). Her family reported that this was accompanied by confusion and lack of logical verbal contact. Apart from confusion, there were no abnormalities in the neurological examination. The differential diagnosis included drug overdose, stroke, sepsis, hypovolemia, hypoglycemia, electrolyte disturbances. Kidney dysfunctions shown in laboratory tests, as well as anemia and proteinuria, prompted doctors to search for rheumatic etiology, which was confirmed in serological tests: positive results for antinuclear and anti-dsDNA antibodies, followed by neuroimaging and CSF examination, which revealed aseptic meningitis. Based on that, NPSLE was diagnosed and initially, methylprednisolone was administered intravenously followed by cyclophosphamide with prednisone p.o. with an improvement both in psychiatric and SLE signs and symptoms (20).

Hirachi et al., on the other hand, described the case of a 35-year-old female, treated for SLE who was admitted to the hospital with the symptoms of SLE exacerbation, which preceded the onset of the episode of mania. The serological test results confirmed the SLE relapse, and the signs and symptoms of mania and SLE were reduced after aripiprazole and steroid therapy, which indicated NPSLE diagnosis (27).

Spiegel et al. mentioned above reported the case of a patient with recurrent episodes of mania in the course of SLE. The authors conducted a differential diagnosis between primary BPD, a clinical syndrome in the course of NPSLE and SIMPD. During the pharmacological treatment, a modest improvement after olanzapine and valproate was observed as well as mental condition deterioration with increasing doses of prednisone, and a marked improvement after cyclophosphamide and low doses of prednisone, which indicated a diagnosis of the episode of mania in the course of NPSLE or SIMPD (21). The differential diagnosis between the episodes of mania and psychosis in the course of NPSLE and SIMPD are shown in Table 3.

**Table 3:** The differential diagnosis between the episodes of mania and psychosis in the course of NPSLE and SIMPD.

	**NPSLE**	**SIMPD**
The disease onset	A year after the diagnosis	2 weeks after administering steroids
Association with steroids	Disease onset after reducing/discontinuing dose of steroids	Dose-dependency
Remission	Months	Less than 3 weeks
Treatment	Steroids and Cyclophosphamide, psychotropic drugs if needed	Scheduled discontinuation, psychotropic drugs if needed
Cerebrospinal fluid examination	Immunological markers (antibodies)	No indications

For this study, the Pubmed and Google Scholar databases were also reviewed using the following keywords: primary bipolar affective disorder, SLE, case report, without obtaining any results. The case report presented here is therefore a unique representation of the co-occurrence of primary BD and SLE.

## Conclusion and the Patient's Perspective

Systemic lupus erythematosus is an autoimmune disease that leads to a chronic inflammatory process in tissues and organs, and NPSLE is one of its variants characterized by the presence of signs and symptoms from the central and peripheral nervous system. Common immunological mechanisms in the course of BD and SLE are the reason for diagnostic difficulties in recognizing BD with comorbid SLE. The differential diagnosis should include primary BD with comorbid SLE, NPSLE, and SIMPD.

According to typical affective symptoms, lack changes in clinical, somatic status of a patient, as well as in laboratory tests, the diagnosis of primary BD with comorbid SLE was set. Due to lack of a patient cooperation in psychotropic drugs administration, sustained-action antipsychotic was prescribed. In the SLE treatment, mycophenolate mofetil and hydroxychloroquine were continued. Months after discharge, the patient presents stable mental status, as well as somatic status according to SLE diagnosis and continues working.

The presented case report is a unique description of a patient with a diagnosis of primary BD with comorbid SLE.

## Data Availability Statement

The original contributions presented in the study are included in the article/supplementary material, further inquiries can be directed to the corresponding author/s.

## Ethics Statement

Ethical review and approval was not required for the study on human participants in accordance with the local legislation and institutional requirements. The patients/participants provided their written informed consent to participate in this study. Written informed consent was obtained from the individual for the publication of any potentially identifiable images or data included in this article.

## Author Contributions

ES and JM contributed to conception and design of the study. JM organized the database. ES wrote the manuscript. Both authors contributed to manuscript revision, read, and approved the submitted version.

## Conflict of Interest

The authors declare that the research was conducted in the absence of any commercial or financial relationships that could be construed as a potential conflict of interest.
